# Spatial Profiling and Prognostic Role of Tumor-Infiltrating CD8+ T and CD20+ B Cells in Metastatic Clear Cell Renal Cell Carcinoma Treated with Sequential Tyrosine Kinase Inhibitors and Nivolumab

**DOI:** 10.7150/jca.125509

**Published:** 2026-01-14

**Authors:** Andriy Trailin, Lenka Červenková, Petr Hošek, Kristýna Pivovarčíková, Michaela Tkadlecová, Petr Stránský, Kari Hemminki, Ondřej Fiala

**Affiliations:** 1 Laboratory of Translational Cancer Genomics, Biomedical Center, Faculty of Medicine in Pilsen, Charles University, Alej Svobody 1665/76, 32300 Pilsen, Czech Republic.; 2 Laboratory of Cancer Treatment and Tissue Regeneration, Biomedical Center, Faculty of Medicine in Pilsen, Charles University, Alej Svobody 1665/76, 32300 Pilsen, Czech Republic.; 3 Department of Pathology, Faculty of Medicine in Pilsen, University Hospital Pilsen, Charles University in Prague, 30460 Pilsen, Czech Republic.; 4 Department of Oncology and Radiotherapeutics, Faculty of Medicine and University Hospital in Pilsen, Charles University, Czech Republic, Alej Svobody 80, 304 60 Pilsen.; 5 Department of Urology, Faculty of Medicine and University Hospital in Pilsen, Charles University, Czech Republic, Edvarda Beneše 1128/13, 301 00 Pilsen, Czech Republic.; 6 Department of Cancer Epidemiology, German Cancer Research Center, Im Neuenheimer Feld 280, 69120 Heidelberg, Germany.; 7 ARON Research Foundation ETS, Macerata, Italy; Galleria del Commercio n. 6, 62100 Macerata, Italy.

**Keywords:** metastatic renal cell carcinoma, progression-free survival, tyrosine kinase inhibitors, immune checkpoint inhibitors, tumor-infiltrating lymphocytes

## Abstract

**Background**: Tumor-infiltrating lymphocytes (TILs) are known to influence disease progression and treatment response in clear cell renal cell carcinoma. This study aimed at evaluating the prognostic and predictive relevance of T and B cell infiltration patterns in patients with metastatic clear cell renal cell carcinoma (mRCC-cc) treated sequentially with tyrosine kinase inhibitors (TKIs) and the immune checkpoint inhibitor nivolumab.

**Methods**: In this retrospective cohort study, immune cell densities (CD3+, CD8+ T cells and CD20+ B cells) were analyzed by immunohistochemistry and quantified using digital image analysis software QuPath in distinct tumor regions of primary tumor: tumor center (TC), inner margin (IM), outer margin (OM), and peritumoral (PT) region. Samples were obtained from 36 patients with mRCC-cc treated with TKIs in the first line and sequentially with nivolumab in the second or third-line setting. Associations between immune cell densities, clinicopathological features, and survival outcomes were assessed using univariable and multivariable Cox regression models. Progression-free survival (PFS), overall survival (OS), and objective response rate (ORR) were evaluated.

**Results**: Densities of all immune cells were significantly higher in the OM and PT regions than in the TC and IM. Older age correlated with lower CD8+ T cell and CD20+ B cell densities, whereas higher tumor grade was associated with increased CD20+ B cell infiltration in IM.

High CD20+ B cell density in IM and OM was significantly associated with shorter PFS during first-line TKI therapy (hazard ratio (HR) = 3.30, P = 0.015 and HR = 3.25, P = 0.016, respectively). In contrast, an intermediate CD8+ T cell density in the PT region was associated with longer PFS during sequential nivolumab treatment (HR = 0.26, P = 0.007). No significant associations between immune cell densities and ORR or OS were observed.

**Conclusions**: Our findings suggest that spatial localization and density of tumor-infiltrating CD20+ B cells are potential predictors of poor PFS on TKIs, whereas higher CD8+ T cell infiltration in peritumoral areas may be a potential predictor of prolonged PFS on nivolumab. These immune-cell-based parameters may refine prognostic models and help guide treatment selection in mRCC-cc.

## Introduction

The global incidence of kidney cancer was estimated at 435,000 cases 1, with 156,000 deaths reported worldwide in 2022 1, 2. Renal cell carcinoma (RCC) accounts for approximately 75-80% of all kidney cancer cases, with clear cell renal cell carcinoma (RCC-cc) being the predominant subtype 3, 4.

Recent data indicate that 15-30% of RCC-cc patients are diagnosed with synchronous metastatic disease 5, while an additional 20-50% eventually develop metachronous metastases 6, 7. Metastatic RCC (mRCC) is a life-threatening condition, with overall survival (OS) typically limited to 2-3 years after diagnosis 8.

Due to high immunogenicity of RCC, which can elicit a robust host immune response 9-11, and also because of tumor resistance to conventional radiotherapy and chemotherapy, the treatment of mRCC is based primarily on targeted therapies and immunotherapies 4, 12.

Among immunotherapeutic options, the anti-PD1 immune checkpoint inhibitor (ICI) nivolumab is frequently used in the treatment of mRCC in various clinical scenarios, such as in combination first-line regimen with cabozantinib or in combination with the anti-CTLA-4 ICI, ipilimumab. As monotherapy, it is used in the second- or third-line setting after failure of VEGF-targeting tyrosine kinase inhibitors (TKIs) 3. Nevertheless, due to tumor heterogeneity and adaptive resistance mechanisms, fewer than half of patients with mRCC respond to TKI therapy 4, 13 or ICI therapy 14, 15.

Therefore, a central focus of immunotherapy research is to elucidate the mechanisms underlying antitumor immune responses and to identify reliable biomarkers that predict response to TKI and ICI therapies 3. In addition to their intrinsic antitumor effects, TKIs also exhibit immune-modulatory properties 16, underscoring the significance of the tumor immune microenvironment (TIME) in mediating therapeutic response. Notably, RCC is characterized by substantial immune cell infiltration, and the composition of its microenvironment significantly influences immunotherapy outcomes 17. However, current prognostic models and treatment strategies remain largely uninformed by host immune characteristics 18.

Depending on the cancer type, tumor-infiltrating lymphocytes (TILs) may either suppress or promote tumor progression 19. In RCC-cc, dense CD8+ T-cell infiltration is often associated with worse prognosis 19, 20; however, favorable prognostic associations have also been reported 21-23, likely reflecting functional heterogeneity among CD8+ T-cell subpopulations 24.

While the role of T lymphocytes has been extensively studied, B cells have historically received less attention in tumor immunology. Nonetheless, increasing evidence implicates tumor-infiltrating B cells in the pathogenesis, prognosis, and therapeutic response in RCC-cc. Existing reports remain contradictory, with some studies linking B cells to tumor promotion 25-29, whereas others suggest antitumor functions 30-32. Similarly, the association between B cells and response to ICI remains unresolved 31, 33.

This study was designed to investigate the prognostic and predictive significance of tumor-infiltrating T and B cells in patients with metastatic clear cell renal cell carcinoma (mRCC-cc) treated sequentially with TKIs in the first-line setting and subsequently with nivolumab in the second or third-line setting.

## Patient and Methods

### Study design and objectives

Immune cell densities in primary tumor tissues were assessed and correlated with baseline clinical parameters and outcomes in consecutive mRCC-cc patients who received systemic therapy. The primary endpoints were progression-free survival (PFS) and objective response rate (ORR) for each therapy. The secondary endpoint was overall survival (OS).

Clinical data were retrospectively extracted from the hospital information system. The study protocol and the informed consent form were approved by the Ethical Committee of the Faculty of Medicine and University Hospital in Pilsen on June 6, 2024 (No. 130/24) and the study was conducted in accordance with the International Ethical Guidelines for Biomedical Research, the Declaration of Helsinki, and local regulations. Written informed consent was obtained from all participants.

### Immunohistochemical staining

Immunoperoxidase detection of CD3+ and CD8+ T cells and CD20+ B cells was performed using a BOND-III IHC/ISH autostainer. Monoclonal primary antibodies to CD3+ (clone LN10) and CD8+ T cells (clone 4B11) and CD20+ (clone L26) were used, all antibodies were obtained from Leica Biosystems (Newcastle Ltd, United Kingdom). After counterstaining with Mayer's hematoxylin, the sections were mounted using Micromount mounting medium (Leica Biosystems Newcastle Ltd., UK). Appropriate negative tissue control samples and positive controls (tonsils) were included throughout.

### Image analysis

Whole-slide scans were acquired using an Olympus VS200 scanner. Open-source image analysis software QuPath (v.0.3.2) was used to make the estimation objective, reliable and reproducible. Using QuPath tools, a border was drawn to separate malignant cell nests from adjacent non-tumor tissue. Regions of interest (ROIs) were defined according to the recommendations of the International Immuno-oncology Biomarkers Working Group 34. The inner invasive margin (IM) and outer invasive margin (OM) were automatically extended as 500 μm-wide regions on each side of the tumor border. The remaining tumor area was annotated as tumor center (TC). The peritumor (PT) region was defined as a 500 μm-wide region adjacent to the OM. Densities of positively stained cells were then determined automatically.

### Data analysis

In addition to densities of individual cell types, CD20+/CD8+ and CD8+/CD3+ ratios were calculated for each ROI. For survival analysis, the raw cell densities and ratios were converted into corresponding percentile values and categorized as low (below the 25^th^ percentile), intermediate (25^th^-70^th^ percentiles) or high (above the 70^th^ percentile) 35.

### Treatment and outcome assessment

Patients were treated in a palliative setting with TKIs (sunitinib or pazopanib) in the first line and sequentially with ICI nivolumab in the second or third line. Sunitinib (Sutent, Pfizer Inc., NYC, New York, USA) was administered orally as a single agent according to standard approved schedules (50 mg/4 weeks on 2 weeks off or 50 mg/2 weeks on 1 week off). Pazopanib (Votrient, Glaxo Smith Kline plc., Brentford, UK) was administered orally as a single agent in the standard approved schedule (800 mg daily). Nivolumab was administered intravenously as a single agent using one of the standard approved schedules (240 mg every two weeks or 480 mg every four weeks). Treatment was continued until disease progression, unacceptable toxicity, or patient refusal. Follow-up visits including physical examination and routine laboratory testing were performed every two to four weeks, and computed tomography imaging was performed every three to four months during treatment. Objective response was assessed locally using Response Evaluation Criteria in Solid Tumors (RECIST) version 1.1 36 and categorized as complete response (CR), partial response (PR), stable disease (SD) and progressive disease (PD). ORR was defined as the proportion of patients achieving CR or PR.

### Statistical analysis

PFS was assessed since the date of initiation of the therapy to the first objective radiologic evidence of disease progression or death from any cause. OS was defined as the time from therapy initiation to death from any cause. PFS and OS were evaluated separately for the first-line TKI therapy and second- or third-line nivolumab therapy. Patients without progression or death at the time of data analysis were censored at the date of last follow-up.

Continuous non-normally distributed variables are presented as median (minimum-maximum); and their comparisons were performed using the Mann-Whitney U Test or Friedman ANOVA, followed by Dunn's test, as appropriate. Categorical variables are expressed as raw data (percentages). Associations between ordinal or quantitative variables were assessed using Spearman correlation.

The prognostic value of individual predictors for PFS and OS was evaluated using univariable Cox regression analysis, followed by multivariable analysis with backward stepwise model construction. Only variables that were significant in the univariable analysis were included in the multivariable model. Hazard ratios (HRs) representing relative risk for the “high” or “intermediate” density group compared with 1 for the “low” group, were calculated.

PFS and OS were estimated using the Kaplan-Meier method and compared between groups by the log-rank test. Statistica 10 (StatSoft Inc, Tulsa, OK, USA) and GraphPad Prism 9.0 (GraphPad Software LLC) were used for the statistical analyses.

A 2-sided *p* value < 0.05 was considered statistically significant.

## Results

### Demographics of RCC patients

Baseline demographic and clinical characteristics of the patients are summarized in Table 1.

All 36 patients included in the study underwent nephrectomy for their RCC-cc and presented with either synchronous distant metastases (47%) or subsequently developed metachronous metastases (53%). The median patient age was 61 years (range 47-78); 67% of patients were males and 33% females. Twenty-eight patients (78%) received sunitinib and eight (22%) received pazopanib as first-line therapy. Subsequently, 16 patients (44%) received second-line TKI therapy (Table 1). Thereafter, all patients were treated with nivolumab as second-line (56%) or third-line (44%) therapy.

The median follow-up time was 76.0 months. Twenty-two patients (61%) had died by the end of follow-up.

### Distribution of TILs in different primary tumor regions

**Figure 1:**
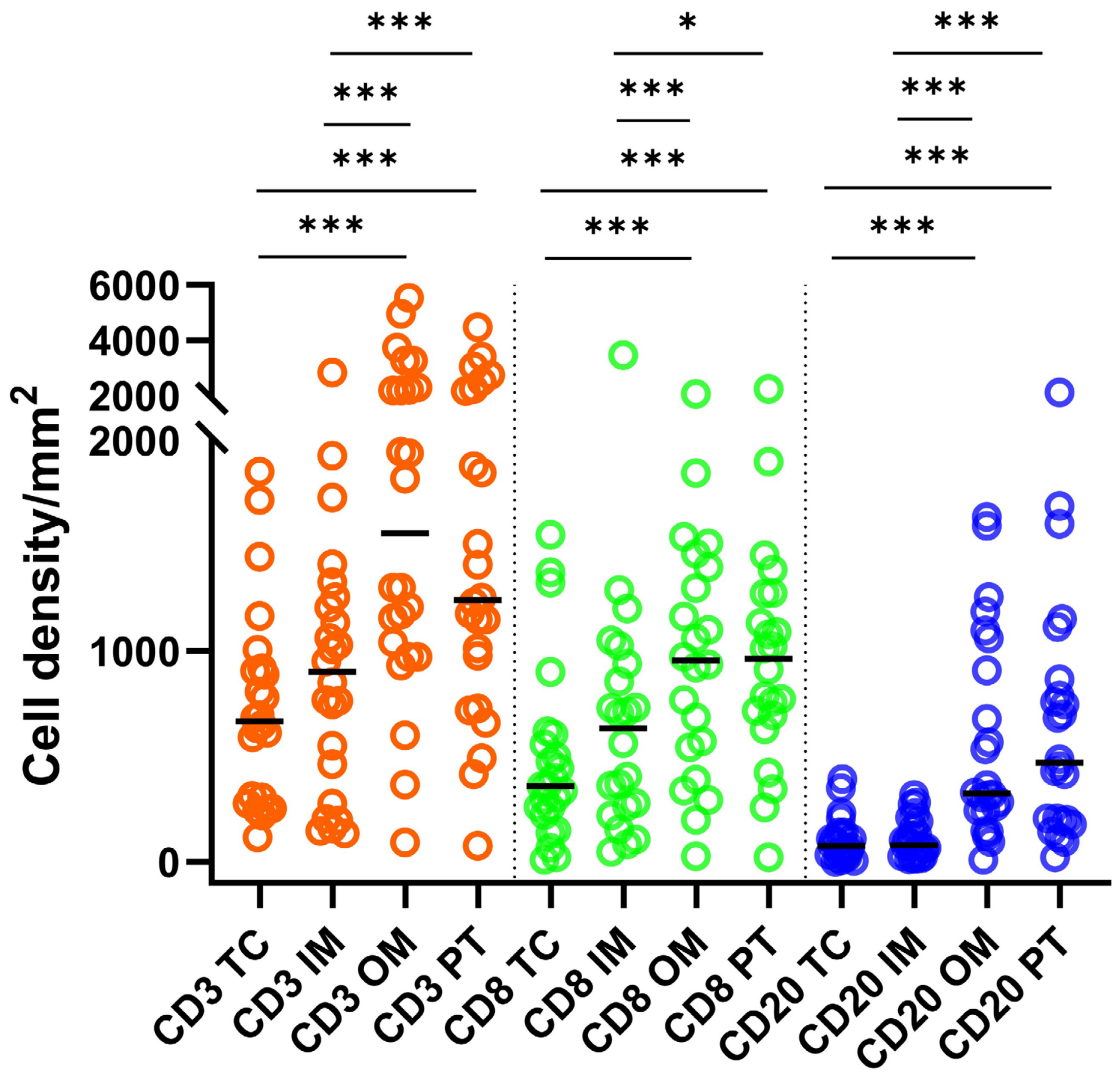
Statistics depicting the spatial distribution of CD3+, CD8+, and CD20+ tumor infiltrating lymphocytes per mm^2^ of the section in the TC, IM, OM, and PT of mRCC-cc. Black lines: medians; *: P < 0.05; ***: P < 0.001. mRCC-cc, metastatic clear cell renal cell carcinoma; IM, inner invasive margin; OM, outer invasive margin; PT, peritumor zone; TC, tumor center.

Densities of CD3+ T cells were greater (P < 0.001) than those of CD8+ and CD20+ cells across all ROIs (Fig. 1). Densities of CD8+ T cells were greater than those of CD20+ cells in the TC, IM (P < 0.001) and OM (P < 0.05). Density of CD3+ and CD8+ T cells and CD20+ B cells in OM and PT regions were significantly greater compared to TC and IM (Fig. 1). A moderate but significant (P < 0.001) correlation was observed between CD8+ and CD20+ cells across all ROIs (Spearmanʼs ρ: 0.64, 0.52, 0.52 and 0.70 for TC, IM, OM and PT region, respectively).

### Associations of TIL densities with clinical-pathological characteristics

Older age at the time of surgery correlated with smaller densities of CD8+ T cells in the IM (ρ = -0.47, P = 0.005), OM (ρ = -0.47, P = 0.02), and PT region (ρ = -0.35, P = 0.04), as well as with smaller densities of CD20+ B cells in TC (ρ = -0.39, P = 0.02) and IM (ρ = -0.38, P = 0.02). Higher tumor grade (grades 3-4) was associated with greater densities of CD20+ B cells (P = 0.047) in the IM compared with lower tumor grade (grades 1-2): 116/mm^2^ (range, 5-318) vs 33/mm^2^ (range, 13-102).

Higher T stage was associated with lower CD8+/CD3+ cell ratios in the IM (ρ = -0.47, P = 0.005) and OM (ρ = -0.38, P = 0.03). No significant differences in immune cell densities were observed in any ROI in relation to tumor stage, gender and synchronicity of metastases (data not shown).

### Survival

In the 1^st^ line of TKI monotherapy, the median PFS and OS were 15.0 months (range, 2.0-79.0) and 50.5 months (range, 9.0-108.0), respectively.

In the 2^nd^/3^rd^ line therapy with nivolumab, the median PFS and OS were 7.0 months (range, 2.0-76.0) and 20.5 months (range, 3.0-76.0), respectively.

PFS and OS estimates at 1, 2, 3 and 4 years are shown in Table S1.

### Association of clinical-pathological characteristics with survival

Male gender was associated with a lower risk of progression during the 1^st^ line TKI therapy: HR = 0.36 (95% confidence interval (CI): 0.17-0.80), P = 0.01. A positive surgical margin (R1) was associated with a higher risk of death after 1^st^ line TKIs therapy: HR = 3.54 (95% CI: 1.18-10.62, P = 0.02) and after 2^nd^ line therapy: HR = 3.46 (95% CI: 1.15-10.38, P = 0.03).

### Associations of TIL densities with first-line TKI survival outcomes

**Figure 2:**
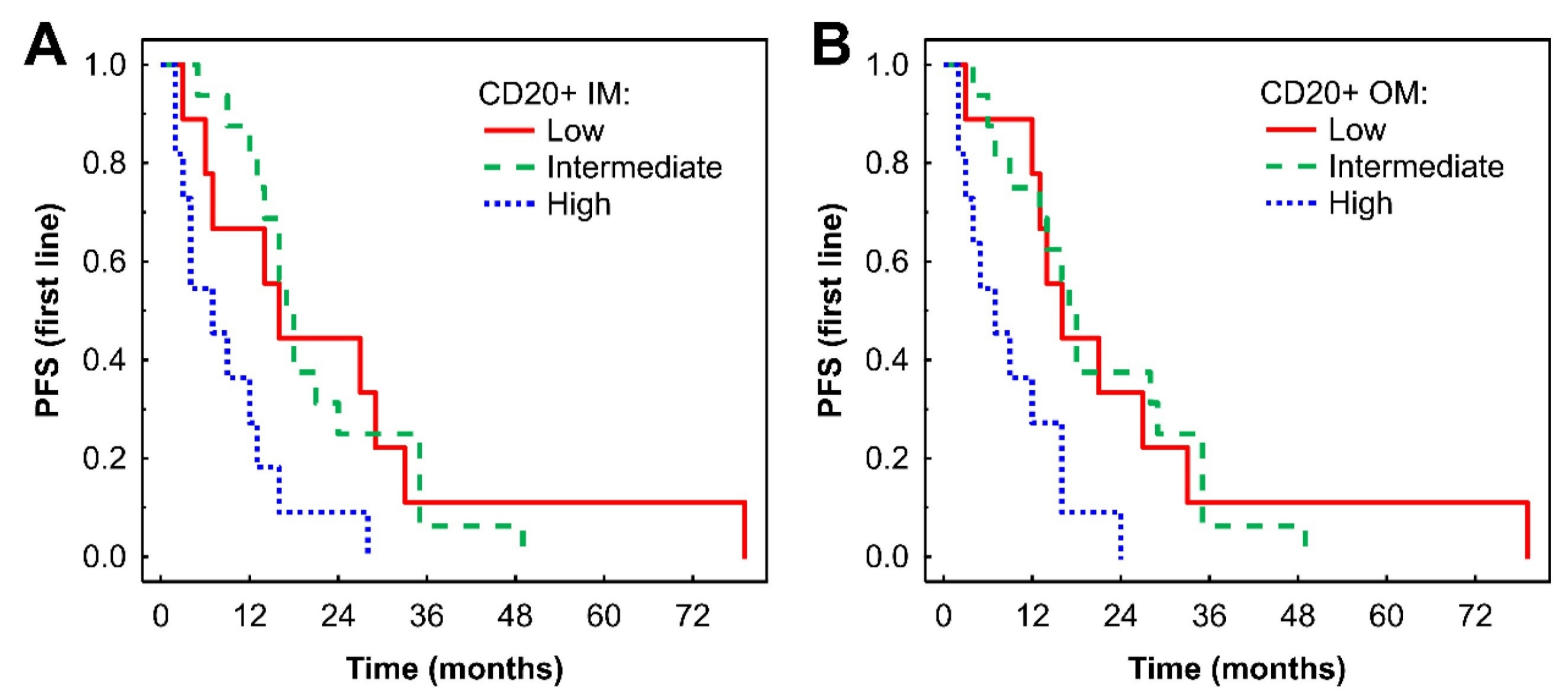
Kaplan-Meier analysis of PFS of mRCC-cc patients on the 1st line of TKI therapy in relation to high, intermediate, and low densities of CD20+ B cells in the inner invasive margin (A) and outer invasive margin (B). PFS, progression-free survival; IM, inner invasive margin; OM, outer invasive margin; mRCC-cc, metastatic clear cell renal cell carcinoma; TKI: tyrosine kinase inhibitors.

In Kaplan-Meier analysis, a high density of CD20+ B cells in the IM was associated with shorter PFS compared with low and intermediate densities (P = 0.033 and P = 0.002, respectively, Fig. 2A). Similar findings were observed in the OM (P = 0.025 and P = 0.004, respectively, Fig. 2B). Subsequent Cox regression analysis confirmed that higher densities of CD20+ B cells in the IM and OM were associated with an increased risk of progression (Table 2). These associations remained significant after adjustment for effects of gender and TKI type: HR = 4.61 (95% CI: 1.67-12.68), P = 0.003 for IM; and HR = 3.26 (95% CI: 1.18-9.01), P = 0.02 for OM.

**Figure 3:**
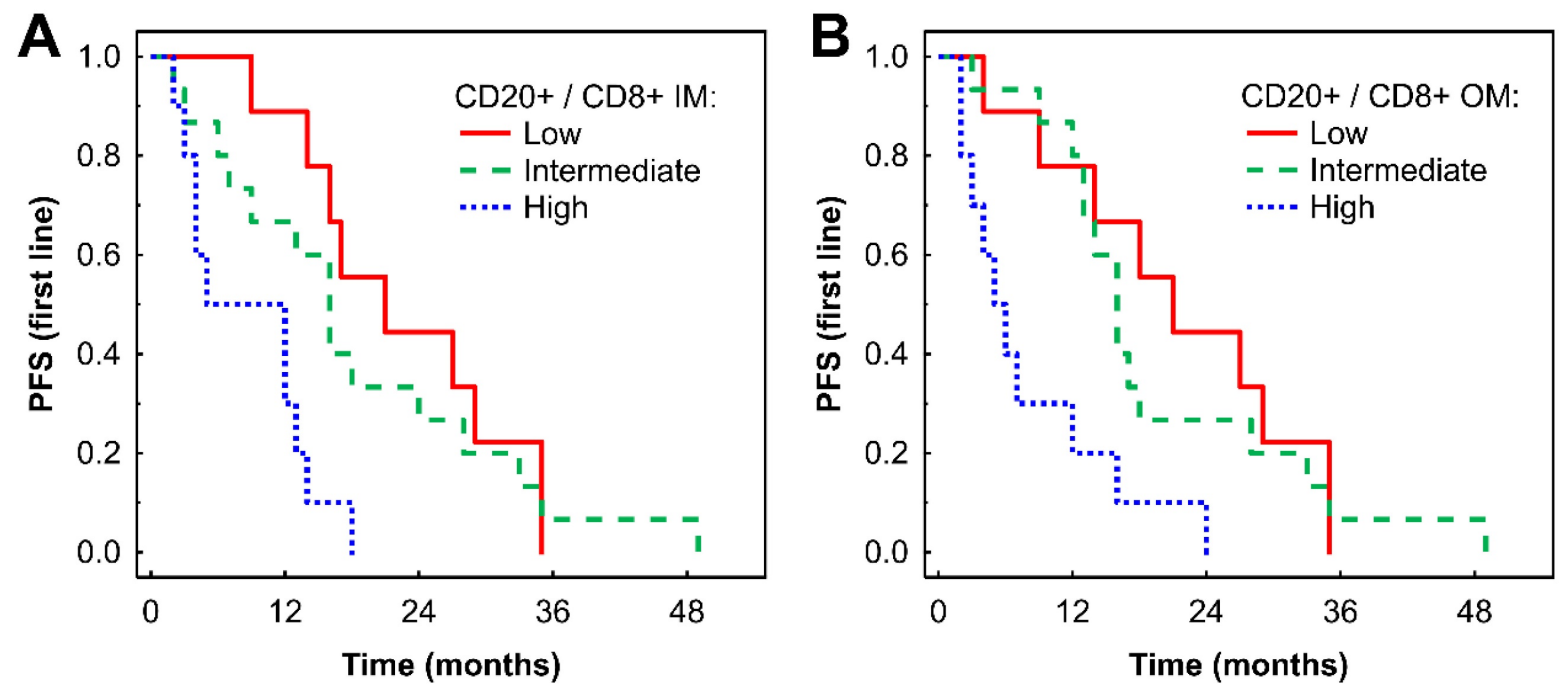
Kaplan-Meier analysis of PFS of mRCC-cc patients on the 1st line of TKI therapy in relation to high, intermediate, and low CD20+/CD8+ ratio in the inner invasive margin (A) and outer invasive margin (B). mRCC-cc: metastatic clear cell renal cell carcinoma; PFS, progression-free survival; IM, inner invasive margin; OM, outer invasive margin; TKI: tyrosine kinase inhibitors.

A high CD20+ to CD8+ ratio in the IM was associated with shorter PFS compared with low and intermediate ratios in Kaplan-Meier analysis (P = 0.001 and 0.015, respectively, Fig. 3A). Similar findings were observed in the OM (P = 0.005 and P = 0.005, respectively, Fig. 3B). These findings for both the IM and OM were confirmed by Cox regression analysis (Table 2). The associations remained significant in a multivariate analysis after adjustment for the effects of gender and TKI type: HR = 3.53 (95% CI: 1.20-10.38), P = 0.022 for CD20+/CD8+ cell ratio in IM; and HR = 3.28 (95% CI: 1.13-9.50), P = 0.028 for CD20+/CD8+ cell ratio in OM.

**Figure 4:**
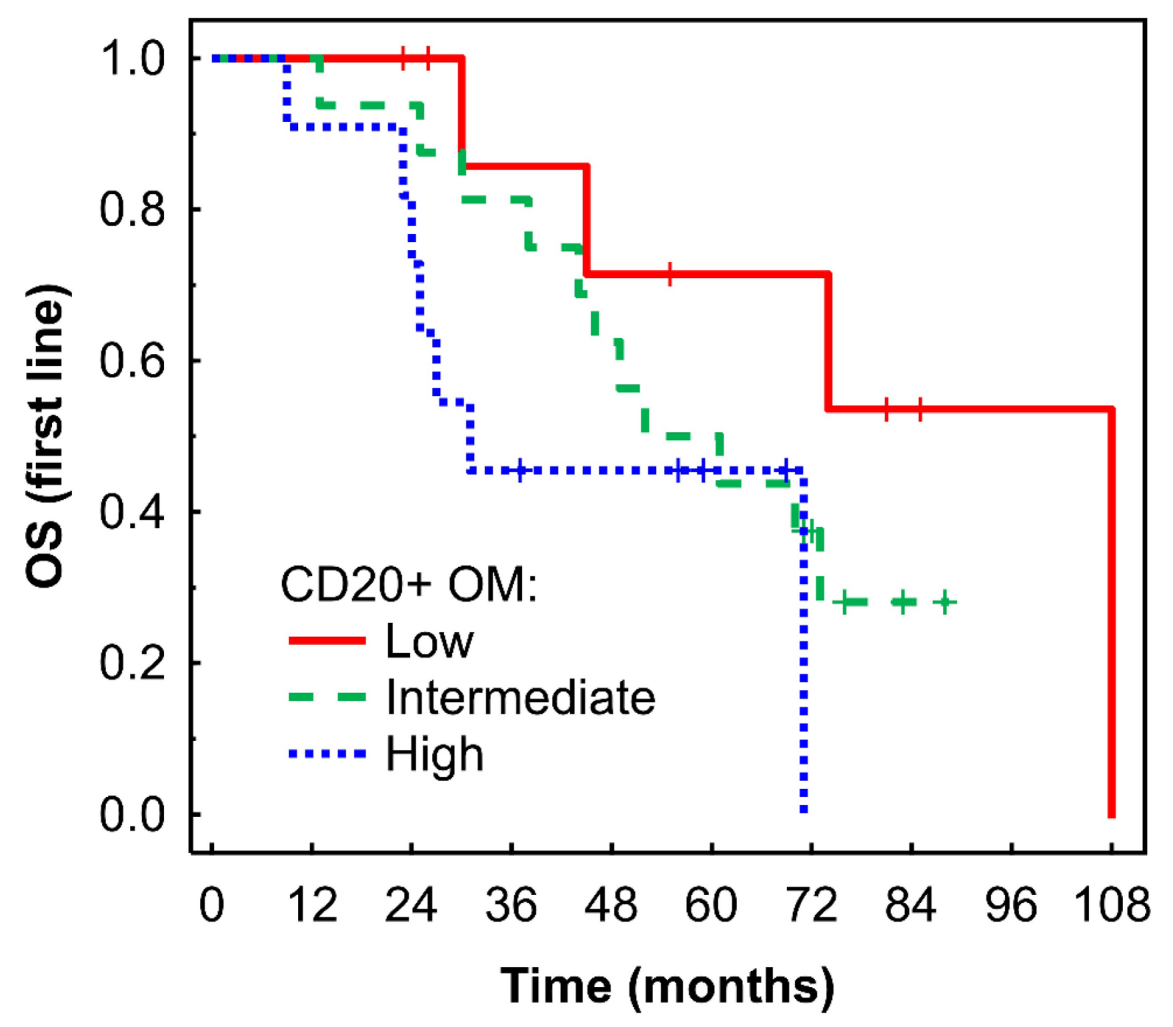
Kaplan-Meier analysis of OS of mRCC-cc patients on the 1^st^ line of TKI therapy in relation to high, intermediate, and low densities of CD20+ B cells in the outer invasive margin. OS, overall survival; OM, outer invasive margin; mRCC-cc: metastatic clear cell renal cell carcinoma; TKI: tyrosine kinase inhibitors.

A high density of CD20+ B cells in the OM was associated with borderline shorter OS in Kaplan-Meier analysis (P = 0.053, Fig. 4) and with a higher risk of death in Cox regression analysis: HR = 3.87 (95% CI: 0.95-15.84), P = 0.060.

### Associations of TIL densities with nivolumab survival outcomes

**Figure 5:**
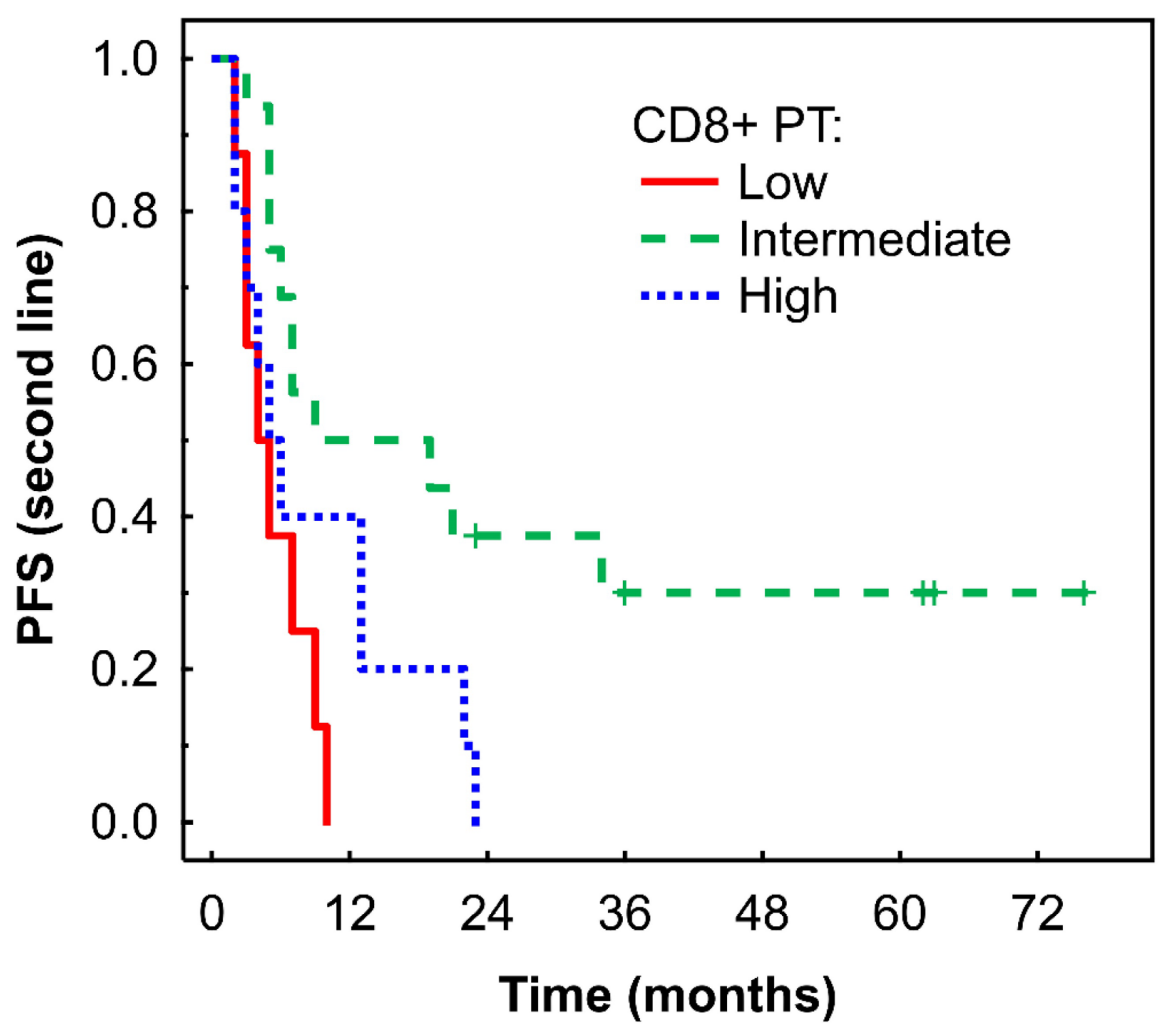
Kaplan-Meier analysis of PFS of mRCC-cc patients on the 2nd line of nivolumab therapy in relation to high, intermediate, and low densities of CD8+ T cells in the PT zone. PFS, progression-free survival; PT, peritumor zone; mRCC-cc: metastatic clear cell renal cell carcinoma.

An intermediate density of CD8+ T cells in the PT region was associated with longer PFS in Kaplan-Meier analysis (P = 0.006, Fig. 5), which was confirmed by Cox regression analysis (Table 2). No significant associations were observed between immune cells or their ratios and OS (data not shown).

### Objective response rate

In the first-line TKI therapy, the ORR was 62%, including 2 patients (6%) with CR and 20 patients (56%) with PR (Table 1); 7 patients (19%) achieved SD and other 7 patients (19%) had PD. The ORR to nivolumab was 31%, including 2 patients (6%) with CR, 9 patients (25%) with PR, 13 patients (36%) with SD, and 12 patients (33%) with PD.

### Associations of TILs densities with objective response rate

Patients who achieved an objective response to first-line TKI therapy or to sequential nivolumab did not show any significant differences in cell densities compared to patients with SD and PD (Tables S2 and S3). However, a trend toward lower densities of CD20+ B cells in the TC: 32 (range, 5-394) vs 95 (range, 1-350), P=0.083) and in the IM: 46 (range, 13-191) vs 132 (range, 5-318), P = 0.077) was observed in patients with objective response compared to those without objective response after 1^st^ line of TKI therapy (Table S2).

## Discussion

This study provides new insights into the spatial distribution and prognostic implications of T and B cells infiltrating mRCC-cc, with a particular focus on prognosis in the context of TKIs and nivolumab therapy. Our findings demonstrate that immune cell composition varies significantly across tumor regions and correlates with clinical outcomes, patient demographics, and tumor biology.

We observed that densities of CD3+ T cells, CD8+ cytotoxic T cells and CD20+ B cells were significantly higher in the OM and PT regions compared to the TC and IM. This gradient suggests an immunological exclusion from the tumor core, a phenomenon well-documented in RCC-cc 37-39, potentially driven by hypoxic or immunosuppressive microenvironments in the tumor center 40-42. However, discordant findings regarding the distribution of T and B cells between tumor and normal tissue have also been reported 43-45.

The predominance of CD8+ T cells over CD20+ B cells in nearly all tumor compartments was reported previously 37, 46, 47 and aligns with the established crucial role of cytotoxic T cells in anti-tumor immunity 48.

Higher tumor grade was associated with increased infiltration of CD20+ B cells in the IM, which may reflect the response of antigen-presenting B cells to neoantigens. Some studies reported increased numbers of intratumor CD8+ T cells in higher grade RCC 37, 43.

The inverse correlation between higher T stage and CD8+/CD3+ ratio in the IM and OM suggests a relative reduction in cytotoxic T cell activity in more advanced tumors, possibly due to T cell exhaustion or recruitment of regulatory T cells 49.

Additionally, aging was associated with a reduction in CD8+ T cell densities in the key immune-active regions, consistent with known immunosenescence and its impact on T cell-mediated anti-tumor responses 50. Older patients also exhibited reduced CD20+ B cell infiltration in the tumor center and inner margin, potentially further compromising local immune surveillance.

A particularly novel and clinically relevant finding is the association of high CD20+ B cell density in the IM and OM with shorter PFS in patients receiving TKIs. This suggests that B-cell-rich tumors may represent a subset with innate resistance to the inhibition of angiogenesis and tumor growth by VEGFR-targeting TKIs. As in other tumors, the TIME in RCC-cc is complex and dynamic, with both immunostimulatory and immunosupressive factors. Considering the dual role of B cells in anti-tumor immune responses reported previously 42, 51, 52, this finding implies a predominance of immunosuppressive functions of B cells. These cells may behave as regulatory B cells that inhibit inflammatory antitumor response, contribute to tumor heterogeneity and promote therapy resistance 53. Regulatory B cells express a variety of check-point molecules, including PD-L1, PD-1 and LAP-TGF-β 28, 29, and can produce immunosuppressive cytokines such as IL-10, TGF-β and IL-35 25-27, which can suppress effector cell responses. These mechanisms may explain our results and the previously reported unfavorable prognostic and predictive associations of high B cell densities in mRCC-cc patients 43.

Potential antitumor effects of B cells reported in the literature, such as antigen-presentation, direct killing of tumor cells, participation in antibody-dependent cellular cytotoxicity, and secretion of anti-tumor cytokines 30, 31, 54, 55, which might have led to improved prognosis in RCC-cc 51, 56, 57 and prediction of TKI benefit 32, appeared to be suppressed in our cohort.

Conversely, patients with intermediate CD8+ T cell density in the PT region experienced longer PFS on nivolumab therapy. This aligns with prior data indicating that moderate T cell infiltration may reflect a more “primed” but not exhausted immune landscape amenable to reinvigoration via PD-1 blockade 58, 59 and with other reports showing favorable predictive and prognostic impact of high CD8+ T cell densities in patients with mRCC-cc receiving nivolumab 21, 22. In particular, in a small study by Shohdy et al. (2024), CD8+ T cells were not associated with PFS on the 1^st^ line TKI therapy, however high CD8+ T cells were associated with longer PFS on 2^nd^ line nivolumab 23.

Contradictory prognostic associations of CD8+ T cells in RCC-cc have been reported: some studies found that CD8+ T cell infiltration correlated with poor prognosis and response to immunotherapy 20, 24, 40, 58, 60, 61, whereas others reported favorable prognostic 57, 62-64 or predictive associations 46, 57, 62. These discrepancies likely reflect functional heterogenity among CD8+ T cell subtypes 24, 65. Within tumor-infltrating CD8+ T cells in RCC-cc, both effector and exhausted phenotypes have been identified 47; the exhaustion phenotype is associated with inferior survival and response to immunotherapy 47. This may explain why total CD8+ T cell densities failed to define the prognosis in several prior RCC-cc studies 37, 43, 45, 66.

Similarly to our findings, Akerla et al. did not find associations between immunoscore and survival in pateints with localized or metastatic RCC-cc 67.

Prognostic associations of B cells were observed in the IM and OM and those of CD8+ T cells in the PT region. These findings illustrate tumor heterogeneity and support the notion that clinically relevant information may be derived not only from the tumor core 66, highlighting the need for refinement of the current guidelines for TIL assessment 34.

The effect of tumor-infiltrating B cells on immunotherapy response is also contradictory. High B cell infiltration improved the prognosis of soft-tissue sarcomas, melanoma, and RCC and predicted a higher response rate to anti-PD-1 therapy 31. However, another study failed to find an association between B cell depletion and response to anti-PD-1 inhibitors in melanoma 33. Therefore, the role of B cells in ICI therapy remains unresolved.

Discrepancies in prognostic and predictive associations of T and B cells between our study and earlier studies may be attributed to insufficient standardization in annotation of tumor regions and quantitative assessment algorithms. In addition, the lack of clear separation of the different histologic RCC subtypes within investigated cohorts may contribute to conflicting findings 60.

### Strengths and limitations of the study

This study has certain limitations, including the relatively small cohort size and the long interval between tumor resection, when the tumor-infiltrating immune cells were evaluated, and subsequent nivolumab treatment. Nevertheless, the study design of evaluating tumor-infiltrating T and B cells in patients receiving front-line TKI monotherapy followed sequentially by nivolumab in the 2^nd^ or 3^rd^ line is novel. Markers used (CD3, CD8 and CD20) do not fully capture the functional heterogeneity of T and B cells. Results would be further strengthened by integrating additional immune cell populations. Our analysis further emphasizes that the prognostic value of T cell infiltration in RCC-cc is context-dependent, varying by T cell subtype and TIME characteristics.

Unlike most studies, we provide a spatially resolved, highly standardized, and reproducible assessment of tumor-infiltrating T and B cells employing digital image analysis according to the state-of-the-art TIL assessment guidelines 34. The use of ubiquitously available IHC staining allows for easy reproduction and potential clinical implementation.

## Conclusion

In conclusion, our results highlight the prognostic significance of spatial profiling of tumor-infiltrating T and B cells in RCC-cc. Future prospective studies and functional analyses are warranted to validate these findings and explore mechanisms underlying the negative prognostic associations of B cells under TKI treatment and positive prognostic impact of CD8+ T cells in patients receiving nivolumab. A deeper understanding of the TIME may be crucial for improved therapeutic decision-making and for tailoring systemic therapy strategies to patient-specific immune landscapes.

## Supplementary Material

Supplementary tables.

## Figures and Tables

**Table 1:** Baseline clinical characteristics of patients.

	Patients (n = 36)
**Sex, n (%)**	
Male	24 (67)
Female	12 (33)
**Age, years**	
Median (Range)	61 (47-78)
**ECOG performance status at nivolumab initiation, n (%)**	
0	12 (33)
1	24 (67)
**IMDC risk group**	
Favorable	4 (11)
Intermediate	28 (78)
NA	4 (11)
**Past nephrectomy, n (%)**	36 (100)
Tumor grade, n (%)	
1-2	10 (28)
3-4	23 (64)
x	3 (8)
**T stage, n (%)**	
1	5 (14)
2	3 (8)
3	26 (72)
4	2 (6)
**Clear cell histology, n (%)**	36 (100)
**Resection margin status**	
R0	22 (61)
R1	5 (14)
Unknown	9 (25)
**Metastatic disease, n (%)**	
Synchronous	17 (47)
Metachronous	19 (53)
**First-line therapy, n (%)**	
sunitinib	28 (78)
pazopanib	8 (22)
**Response to 1^st^ line TKI therapy, n (%)**	
CR/PR	22 (62)
SD	7 (19)
PD	7 (19)
**Second-line therapy, n (%)**	
none	20 (56)
sunitinib	1 (3)
axitinib	7 (19)
cabozantinib	8 (22)
**Nivolumab therapy, n (%)**	
Second line	20 (56)
Third line	16 (44)
**3rd lines of nivolumab therapy, n (%)**	
CR/PR	11 (31)
SD	13 (36)
PD	12 (33)

Abbreviations: ECOG: Eastern Cooperative Oncology Group; IMDC: International Metastatic RCC Database Consortium; TKI: tyrosine kinase inhibitor; CR: complete response; PR: partial response; SD: stable disease; PD: progressive disease.

**Table 2:** Hazard ratios for progression-free survival between high or intermediate versus low T- and B-cell cell density per individual ROI in mRCC-cc patients under the TKIs and nivolumab therapy.

	PFS on TKIs	PFS on nivolumab
**CD3 TC**		
intermediate	0.98 (0.42-2.31), P = 0.97	0.62 (0.26-1.47), P = 0.28
high	0.93 (0.37-2.35), P = 0.88	0.76 (0.31-1.89), P = 0.56
**CD3 IM**		
intermediate	1.04 (0.44-2.46), P = 0.92	0.81 (0.35-1.91), P = 0.63
high	1.33 (0.53-3.46), P = 0.55	0.69 (0.27-1.74), P = 0.43
**CD3 OM**		
intermediate	0.97 (0.41-2.29), P = 0.94	0.88 (0.38-2.05), P = 0.77
high	1.60 (0.62-4.15), P = 0.33	0.60 (0.23-1.57), P = 0.30
**CD3 PT**		
intermediate	0.95 (0.40-2.24), P = 0.91	0.41 (0.17-1.02), P = 0.056
high	2.27 (0.88-8.88), P = 0.09	0.63 (0.25-1.61), P = 0.33
**CD8 TC**		
intermediate	2.02 (0.82-4.99), P = 0.13	1.05 (0.42-2.60), P = 0.92
high	1.41(0.54-3.64), P = 0.48	1.16 (0.44-3.05), P = 0.77
**CD8/CD3 TC**		
intermediate	0.85 (0.37-1.96), P = 0.71	1.14 (0.46-2.82), P = 0.78
high	0.93 (0.38-2.29), P = 0.87	1.09 (0.41-2.86), P = 0.86
**CD8 IM**		
intermediate	0.56 (0.24-1.35), P = 0.20	1.69 (0.67-4.27), P = 0.27
high	0.62 (0.24-1.60), P = 0.32	0.94 (0.34-2.60), P = 0.90
**CD8/CD3 IM**		
intermediate	0.80 (0.34-1.91), P = 0.62	0.76 (0.31-1.84), P = 0.54
high	0.84 (0.33-2.14), P = 0.72	0.68 (0.26-1.84), P = 0.45
**CD8 OM**		
intermediate	0.78 (0.33-1.85), P = 0.57	0.47 (0.19-1.21), P = 0.12
high	0.90 (0.35-2.34), P = 0.83	0.74 (0.28-1.93), P = 0.53
**CD8/CD3 OM**		
intermediate	0.84 (0.36-2.01), P = 0.70	0.80 (0.32-1.96), P = 0.62
high	0.67 (0.26-1.72), P = 0.41	0.86 (0.33-2.23), P = 0.75
**CD8 PT**		
intermediate	1.46 (0.60-3.59), P = 0.41	0.26 (0.10-0.69), **P = 0.007**
high	1.35 (0.51-3.55), P = 0.55	0.63 (0.24-1.67), P = 0.35
**CD8/CD3 PT**		
intermediate	1.48 (0.63-3.50), P = 0.37	1.02 (0.40-2.55), P = 0.98
high	0.98(0.39-2.51), P = 0.97	1.18 (0.44-3.18), P = 0.74
**CD20 TC**		
intermediate	1.07 (0.46-2.51), P = 0.87	1.05 (0.43-2.54), P = 0.92
high	1.90 (0.75-4.78), P = 0.18	1.05 (0.41-2.68), P = 0.91
**CD20/CD8 TC**		
intermediate	0.79 (0.34-1.85), P = 0.59	0.71 (0.29-1.75), P = 0.46
high	2.21 (0.87-5.70), P = 0.10	1.24 (0.50-3.09), P = 0.65
**CD20 IM**		
intermediate	1.00 (0.43-2.37), P = 0.99	0.62 (0.26-1.45), P = 0.27
high	3.30 (1.27-8.62), **P = 0.015**	0.78 (0.30-1.98), P = 0.60
**CD20/CD8 IM**		
intermediate	1.29 (0.561-2.98), P=0.56	1.02 (0.42-2.47), P = 0.96
high	4.46 (1.64-12.97), **P = 0.003**	0.80 (0.30-2.15), P = 0.66
**CD20 OM**		
intermediate	0.99 (0.42-2.34), P = 0.99	1.02 (0.43-2.43), P = 0.97
high	3.25 (1.24-8.50), **P = 0.016**	1.03 (0.40-2.68), P = 0.95
**CD20/CD8 OM**		
intermediate	1.19 (0.51-2.76), P = 0.67	1.81 (0.73-4.52), P = 0.20
high	4.15 (1.57-10.97), **P = 0.004**	1.09 (0.40-3.02), P = 0.86
**CD20 PT**		
intermediate	1.05 (0.45-2.47), P = 0.91	0.53 (0.21-1.30), P = 0.17
high	1.58 (0.63-3.98), P = 0.33	0.57 (0.22-1.47), P = 0.25
**CD20/CD8 PT**		
intermediate	0.58 (0.25-1.35), P = 0.21	1.26 (0.51-3.14), P = 0.62
high	2.31 (0.89-6.01), P = 0.09	0.80 (0.29-2.23), P = 0.68

Abbreviations: TC: tumor center; IM: inner invasive margin; OM: outer invasive margin; PT: peritumor region; ROIs: regions of interest; mRCC-cc: metastatic clear cell renal cell carcinoma; PFS, progression-free survival; TKI: tyrosine kinase inhibitors
