# Supplementary material for: Spatial Profiling and Prognostic Role of Tumor-Infiltrating CD8+ T and CD20+ B Cells in Metastatic Clear Cell Renal Cell Carcinoma Treated with Sequential Tyrosine Kinase Inhibitors and Nivolumab
Source: J Cancer. 2026 Jan 14;17(2):372–81. doi: 10.7150/jca.125509 (PMC12825419; doi:10.7150/jca.125509)
Supplement: Supplementary file 1 — Supplementary tables. [file jcav17p0372s1.pdf]

Supplementary materials

Table S1. The estimated probability of outcomes in Kaplan-Meier analysis

	1 year	2 years	3 years	4 years
PFP, 1st line of TKIs	61.11% (45.19–77.04)	25.00% (10.85–39.15)		
OS, 1st line of TKIs	95.14% (88.18–102.09)	88.80% (78.46–99.15)	68.99% (53.52–84.46)	56.76% (39.98–73.54)
PFP on nivolumab in the 2nd/3rd lines	32.41% (17.15–47.67)	16.36% (4.26–28.46)		
OS on nivolumab in the 2nd/3rd lines	77.12% (63.16–91.08)	46.76% (29.76–63.76)	39.08% (21.78–56.37)	28.71% (9.82–47.59)

Abbreviations: PFP: progression-free proportion; OS: overall survival; TKI: tyrosine kinase inhibitors.

Table S2. Densities of tumor-infiltrating T and B cells in mRCC-cc patients with respect to objective response to TKI therapy in the 1st line

	No OR (N=14)	OR (N=22)	P-value
CD3 TC	740 (84-1951)	602 (117-1578)	0.57
CD3 IM	980 (64-1928)	758 (136-3461)	0.34
CD3 OM	1883 (181-5537)	1440 (93-3362)	0.49
CD3 PT	1836 (416-4480)	1242 (75-2508)	0.25
CD8 TC	392 (22-1553)	318 (22-1557)	0.89
CD8 IM	563 (64-1201)	406 (47-2829)	0.83
CD8 OM	923 (180-2914)	972 (26-1846)	0.94
CD8 PT	900 (423-2239)	772 (22-1494)	0.40
CD20 TC	95 (1-350)	32 (5-394)	0.08
CD20 IM	132 (5-318)	46 (13-191)	0.08
CD20 OM	634 (96-2125)	276 (10-1460)	0.17
CD20 PT	569 (95-2110)	452 (22-1218)	0.27

Abbreviations: OR: objective response; TC: tumor center; IM: inner margin; OM: outer margin; PT: peritumor region. mRCC-cc: metastatic clear cell renal cell carcinoma; TKI: tyrosine kinase inhibitors.

Table S3. Densities of tumor-infiltrating T and B cells in mRCC-cc patients with respect to objective response to nivolumab therapy in the 2nd or 3rd lines

	No OR (N=25)	OR (N=11)	P-value
CD3 TC	634 (84-1951)	698 (248-1852)	0.59
CD3 IM	827 (64-1414)	1030 (136-3461)	0.31
CD3 OM	1300 (93-5451)	2190 (602-5537)	0.34
CD3 PT	1230 (75-3436)	1594 (416-4480)	0.56
CD8 TC	337 (22-1557)	401 (22-1553)	0.87
CD8 IM	369 (47-1412)	856 (157-2829)	0.14
CD8 OM	916 (26-2914)	1182 (605-1543)	0.16
CD8 PT	760 (22-1920)	1021 (562-2239)	0.22
CD20 TC	53 (5-394)	46 (1-350)	1.00
CD20 IM	63 (5-318)	73 (13-281)	0.61
CD20 OM	285 (10-2125)	365 (122-1637)	0.59
CD20 PT	454 (22-1690)	487 (144-2110)	0.74

Abbreviations: OR: objective response; TC: tumor center; IM: inner margin; OM: outer margin; PT: peritumor region; mRCC-cc: metastatic clear cell renal cell carcinoma
